# Architecture of high-affinity unnatural-base DNA aptamers toward pharmaceutical applications

**DOI:** 10.1038/srep18478

**Published:** 2015-12-22

**Authors:** Ken-ichiro Matsunaga, Michiko Kimoto, Charlotte Hanson, Michael Sanford, Howard A. Young, Ichiro Hirao

**Affiliations:** 1 Institute of Bioengineering and Nanotechnology, 31 Biopolis Way, The Nanos, #04-01, Singapore 138669; 2 TagCyx Biotechnologies, 1-6-126 Suehiro-cho, Tsurumi-ku, Yokohama, Kanagawa 230-0045, Japan; 3 RIKEN Center for Life Science Technologies, 1-7-22 Suehiro-cho, Tsurumi-ku, Yokohama, Kanagawa 230-0045, Japan; 4 PRESTO, JST, Honcho, Kawaguchi-shi, Saitama 332-0012, Japan; 5 Cancer and Inflammation Program, National Cancer Institute at Frederick, Frederick, MD, 21702 USA

## Abstract

We present a remodeling method for high-affinity unnatural-base DNA aptamers to augment their thermal stability and nuclease resistance, for use as drug candidates targeting specific proteins. Introducing a unique mini-hairpin DNA provides robust stability to unnatural-base DNA aptamers generated by SELEX using genetic alphabet expansion, without reducing their high affinity. By this method, >80% of the remodeled DNA aptamer targeting interferon-γ (*K*_D_ of 33 pM) survived in human serum at 37 °C after 3 days under our experimental conditions, and sustainably inhibited the biological activity of interferon-γ.

In clinical medicine, neutralizing antibodies have revolutionized the treatments of numerous diseases and improved the quality of life for thousands of patients. However, these antibodies occasionally evoke undesirable immune responses to themselves, and quality control in mass production can be problematic^1^. Thus, DNA aptamers represent an alternate approach for targeting proteins^2^,^3^,^4^.

DNA aptamers are oligonucleotides that bind to a variety of targets, such as small molecules, oligosaccharides, peptides, proteins, and cells. They are generated by an evolutionary engineering method called SELEX, using an oligonucleotide library with a randomized sequence^5^,^6^. Once DNA aptamer sequences are obtained by SELEX, the aptamers can be chemically synthesized on a large GMP scale, for diagnostic and therapeutic applications. Nevertheless, no DNA aptamer drugs have been approved yet for clinical use. Only a modified RNA aptamer (pegaptanib sodium, Macugen) against vascular endothelial cell growth factor-165 (VEGF165) has been approved, as a treatment for neovascular age-related macular degeneration^7^.

As clinical candidates, generated DNA or RNA aptamers are initially screened by their biological activity, based on target affinity, thermal stability, and nuclease resistance. However, the *K*_D_ values of most aptamers generated by SELEX are around nM orders, and the aptamers often lack biological activity. Another problem is their low stability against nucleases, and thus the nucleotide components in aptamers must be protected by chemical modifications^4^,^8^,^9^,^10^,^11^,^12^, such as 2′-methoxy- and/or 2′-halide-ribonucleotides or 3′-bioconjugated oligonucleotides, which often reduce their affinity and/or increase the production cost. Other versions include RNA aptamers bearing L-ribose with increased nuclease resistance, as reported by Spiegelmer^13^, and DNA aptamers containing modified bases, such as SOMAmer^14^ and X-aptamer^15^, with improved aptamer generation success rates. However, with these methods, the significant augmentation of the aptamer affinities to their targets is still an intractable problem.

We recently developed a new SELEX method to generate high-affinity DNA aptamers, by applying genetic alphabet expansion using an artificial extra base pair (unnatural base pair)^16^,^17^,^18^ between the hydrophobic Ds and Px bases that functions in PCR as a third base pair (SELEX procedure in Fig. 1a). The *K*_D_ values of Ds-containing DNA aptamers generated by this method are lower than pM for VEGF165 and 46 ± 8 pM for interferon-γ (IFNγ)^18^. We now report the high biological activity of the anti-IFNγ DNA aptamer in cell culture experiments, and an aptamer remodeling method that greatly increases the thermal stability and nuclease resistance (Fig. 1). The method could open the door to new unnatural-base DNA aptamer drug candidates for clinical trials.

## Results

### Biological activity of the anti-IFNγ DNA aptamer

We confirmed that the high affinity of the aptamers to their targets is essential for their biological activity, by inhibition experiments using the unnatural-base DNA aptamer against IFNγ, a cytokine that induces STAT1 phosphorylation in cultured cells^19^ (Fig. 2a). The mixture of recombinant human IFNγ (2 ng/ml, ~118 pM as a monomer) and the anti-IFNγ DNA aptamer (50–200 ng/ml, Fig. 3) was added to the human breast tumor cell line in 10% fetal calf serum, and incubated at 37 °C for 10 min. The cells were stained intracellularly, using an anti-phospho-STAT1 antibody and FITC-conjugated F(ab′)_2_ goat anti-rabbit IgG, and detected by flow cytometry (FACS). The addition of 50 ng/ml (~3 nM) of the original anti-IFNγ DNA aptamer^18^ (49-mer, aptamer 49, *K*_D_ = 46 pM) completely inhibited the STAT1 phosphorylation (Fig. 2b). In contrast, a conventional DNA aptamer consisting of only the natural bases had no effect (26-mer, aptamer 26, *K*_D_ = 16 nM)^20^ (Fig. 2c). Replacing three Ds bases in aptamer 49 with A (aptamer 49A, *K*_D_ = 7.2 nM) also had no inhibitory effect (Fig. 2d). These results revealed that the low *K*_D_ values (subnanomolar) are the first threshold for aptamers to exhibit their biological activity and be considered as drug candidates.

The next barrier toward the pharmaceutical application of Ds-containing DNA aptamers is their stability against nucleases. The Ds-containing DNA aptamers are degraded in serum. For example, aptamer 49 was mostly degraded after 24 hours at 37 °C in human serum (Fig. 4a), and lost the activity in an overnight assay (Fig. 2e). The degradation of aptamer 49 was as high as those of single-stranded DNA fragments consisting of only natural bases (control DNA: ssDNA1 and ssDNA2 50-mer), although a small amount of partially digested fragments of aptamer 49 seemed to remain, as compared to the control DNA fragments (Supplementary Fig. 1). This slight nuclease resistance of aptamer 49 might result from its tertiary structure formation, rather than the incorporated Ds bases. The relatively low thermal stability of aptamer 49 (Thermal melting temperature (*T*_m_) = 37.8 °C) (see Supplementary Fig. 2) might also affect the degradation.

### Remodeling the anti-IFNγ DNA aptamer by introducing a mini-hairpin DNA

**Figure 1:**
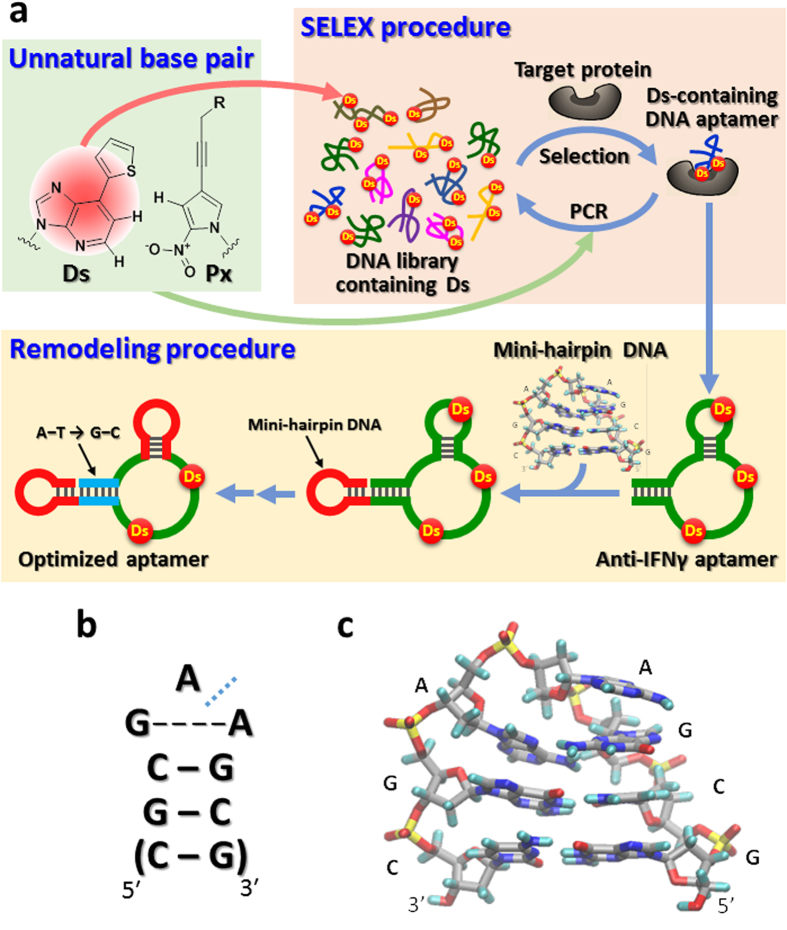
Unnatural-base DNA aptamer generation and remodeling toward diagnostic and therapeutic applications. (**a**) Generation of unnatural-base DNA aptamers by SELEX involving the Ds–Px pair, and aptamer remodeling strategy by introducing mini-hairpin DNA. The original aptamer sequences are colored green, the mini-hairpin regions are red, and the stem region with A–T replaced with G–C is blue. (**b**) The secondary structure of an extraordinarily stable mini-hairpin DNA with a GAA loop and the consecutive two or three G–C base pairs as the stem region. The blue dashed line between the two As indicates the kinked position of the GCGAAGC fragment. A non-canonical shared G–A base pair is formed in the GAA loop. (**c**) The tertiary structure of the mini-hairpin DNA, obtained by NMR spectroscopy^22^.

To address the aptamer degradation, we introduced an extraordinarily stable mini-hairpin DNA into the anti-IFNγ DNA aptamer. Previously, we discovered unique mini-hairpin DNA sequences consisting of a GNA or GNNA loop (N = A, G, C or T) and at least two G–C pairs (Fig. 1b and 1c)^21^,^22^,^23^. The *T*_m_ value of a GCGAAGC mini-hairpin DNA fragment is as high as 78 °C, and the mini-hairpin DNA fragments are remarkably resistant to nucleases. Furthermore, attaching the mini-hairpin DNA fragment to the 3′-terminus of other oligonucleotides endows resistance against 3′-exonucleases^24^.

Aptamer 49 was remodeled, according to the scheme shown in Fig. 1a (Remodeling procedure), Fig. 3, and Supplementary Table 1. First, a mini-hairpin DNA, CGCGAAGCG, including three G–C pairs and a GAA loop was attached to the 3′-terminus of aptamer 49 (aptamer 58), for resistance to the 3′-exonucleases. Second, the mini-hairpin DNA was replaced with the internal hairpin structure which might not involve the direct interaction with its target (aptamer 57 mh), to stabilize the entire aptamer tertiary structure and confer endonuclease resistance. Our previous studies of aptamer 49 showed that this hairpin loop sequence including Ds is less conserved for the binding^18^. Third, all of the A–T pairs in the terminal stem region were replaced with G–C pairs (aptamer 57 mhGC), to further stabilize the aptamer structure. We presumed that the two Ds bases directly interact with IFNγ, and other stem or hairpin regions might reinforce the stabilization of the aptamer scaffold.

The high potential of this remodeling strategy was confirmed by the significantly enhanced stability of the aptamer without affinity reduction, as well as the increased endurance of the inhibitory activity to IFNγ (Fig. 4). The nuclease resistance and the thermal stability of the remodeled aptamers were improved step by step. In particular, the *T*_m_ value of aptamer 57 mhGC (*T*_m_ = 64.2 °C) increased by ~26 °C, relative to that of aptamer 49 (*T*_m_ = 37.8 °C) (Fig. 4 and Supplementary Fig. 2). From the first derivations of the *T*_m_ measurements, the characteristic melting profiles of the mini-hairpin regions of aptamer 57 mh and aptamer 57 mhGC were observed at 85–90 °C (Supplementary Fig. 2). In addition, the *T*_m_ curvature of aptamer 57 mhGC was broadened, as compared to the other profiles. Thus, the tertiary structure of aptamer 57 mhGC might melt gradually at certain regions. Importantly, the *T*_m_ profiles indicated that aptamer 57 mhGC is much more stable at around 37 °C, relative to the other aptamers. This observation was confirmed by the increased nuclease resistance of the remodeled aptamers (Fig. 4 and Supplementary Fig. 3). Finally, more than 80% of aptamer 57 mhGC survived after an incubation in serum at 37 °C for 3 days.

This remodeling did not affect the aptamer affinity to targets, and the *K*_D_ value of aptamer 57 mhGC (*K*_D_ = 33 pM) was as high as that of aptamer 49 (*K*_D_ = 46 pM) (Fig. 4). The chi square values for the determination of the *K*_D_ values using a 1 : 1 complex were relatively large, but were reduced by the remodeling process (Supplementary Fig. 4). The chi square values of aptamer 49 were 2.7–4.9 using a 1 : 1 complex and 0.6–0.7 using a heterogeneous ligand complex, indicating the possibility that aptamer 49 adopts two structures, a major one with high affinity (pM order *K*_D_ value) and a minor one with low affinity (nM order *K*_D_ value). In contrast, the chi square value of aptamer 57 mhGC using a 1 : 1 complex was improved (0.6–1.3). Thus, our remodeling method to increase the rigidity of the stem regions might unify the tertiary structure of the aptamer and enhance its stability, without reducing its affinity to targets.

**Figure 2:**
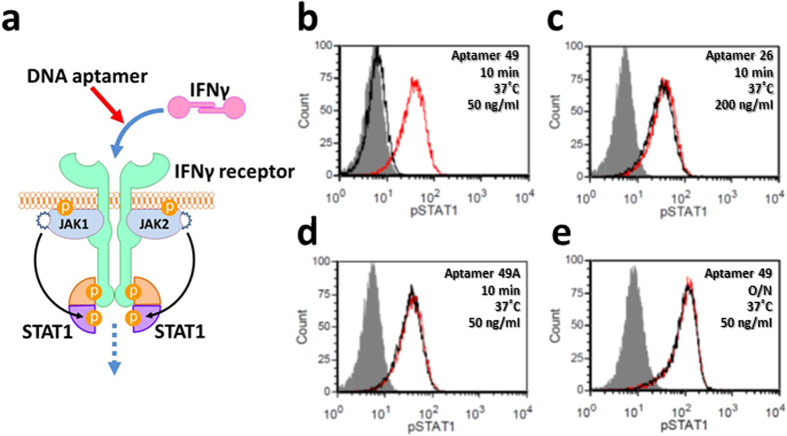
Inhibition of the interaction between IFNγ and its receptor by anti-IFNγ DNA aptamers. (**a**) Schematic illustration of the inhibition of the IFNγ-induced cellular signalling pathway by an anti-IFNγ DNA aptamer. (**b–e**) FACS analysis of STAT1 phosphorylation in the presence of each aptamer. Black and red peaks represent 2 ng/ml of human IFNγ treatment with and without aptamers (**b**) 50 ng/ml of aptamer 49 for 10 min, (**c**) 200 ng/ml of aptamer 26 for 10 min, (**d**) 50 ng/ml of aptamer 49A for 10 min, (**e**) 50 ng/ml of aptamer 49 overnight), respectively. Grey peaks represent unstimulated cells.

**Figure 3:**
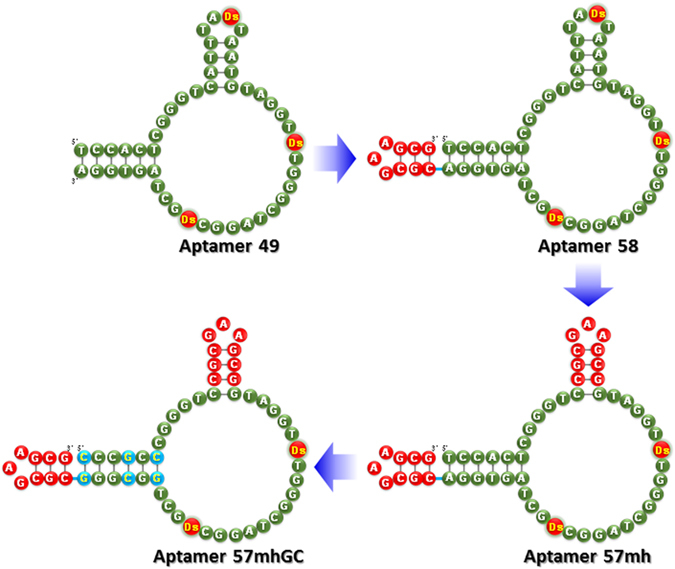
Remodeling strategy of the anti- IFNγ DNA aptamer from aptamer 49 to aptamer 57 mhGC. The remodeled regions are indicated in red circles with white letters (CGCGAAGCG) and blue circles with yellow letters (A–T replaced with G–C).

**Figure 4:**
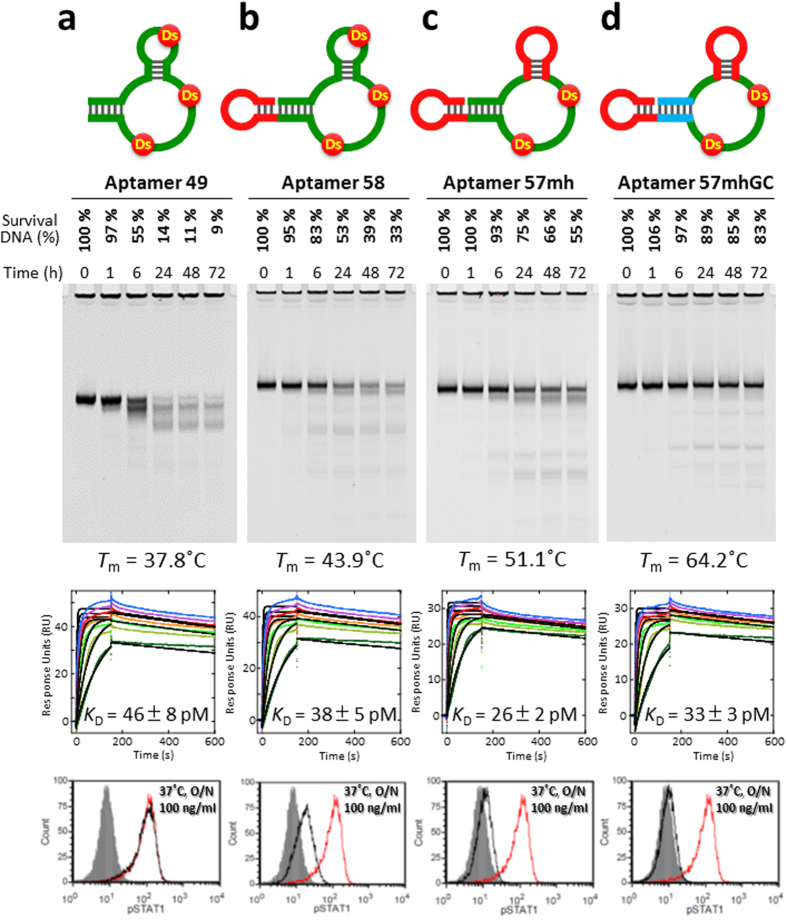
Characterization of the original and remodeled unnatural-base DNA aptamers. (**a**) Original and (**b–d**) remodeled anti-IFNγ DNA aptamers (**a**) aptamer 49, (**b**) aptamer 58, (**c**) aptamer 57 mh, (**d**) aptamer 57 mhGC), characterized by nuclease resistance in 96% human serum at 37 °C (Supplementary Fig. 3), thermal stability (Supplementary Fig. 2), binding ability analyzed by BIAcore T200 in the presence of 1.25–50 nM IFNγ (Supplementary Fig. 5), and STAT1 phosphorylation for the inhibition of IFNγ (FACS analysis). Black and red peaks represent human IFNγ treatment with and without aptamers, respectively. Grey peaks represent unstimulated cells.

The biological activity was also improved step by step by the remodeling (Fig. 2d, Supplementary Fig. 5). The IFNγ inhibition using 100 ng/ml (~6.2 nM) of aptamers was clearly enhanced by the remodeling. In particular, aptamer 57 mhGC completely inhibited the IFNγ activity after an overnight incubation at 37 °C (Fig. 4d), and even after 3 days (Supplementary Fig. 6).

## Discussion

We have presented the remodeling of high-affinity unnatural-base DNA aptamers, which function as alternatives to antibodies. The remodeled aptamer 57 mhGC targeting IFNγ, in cases where cytokine expression directly contributes to disease pathogenesis^25^, might be applied to diagnostics and therapeutics. This remodeling process revealed that sub-nM *K*_D_ values, as measured by surface plasmon resonance (SPR), might be a pivotal requirement for the use of nucleic acid aptamers in diagnostic and therapeutic applications. To this end, the thermal stabilities of the tertiary structures of unnatural-base DNA aptamers must be increased, to confer nuclease resistance at a physiological temperature. Introducing the Ds bases greatly augmented the aptamer affinity to targets, and remodeling using the mini-hairpin DNA significantly enhanced the thermal stability and nuclease resistance.

Unlike conventional chemical modifications of aptamers, our method using natural oligonucleotides, containing a few low-toxicity Ds bases^26^, has a profound effect on aptamer stability, and thus will provide low-cost drug candidates with minimal toxicity. In addition, the mini-hairpin DNA linkage to the 3′-terminus of the terminal stem region of the aptamers stabilizes them more efficiently, as compared to the conventional 3′-3′ inverted dT modification used in pegaptanib sodium for the protection of the 3′-terminus of the aptamer against 3′-exonucleases. The 3′-terminal base of the mini-hairpin DNA also protects the 5′-terminus of the aptamer, by base stacking with the 5′-base of the aptamer (data not shown).

For this remodeling, information about the secondary structures of the aptamers is essential, and can be obtained by doped selection using a library with partially randomized aptamer sequences^18^. Although the base sequences in the stem-loop regions that are essential for direct binding cannot be replaced with mini-hairpin sequences, most of the A–T pairs in the stem regions of aptamers might be replaced with G-C pairs. This is because the stem regions in the Ds-containing DNA aptamers are important as a scaffold to facilitate the interaction of the hydrophobic Ds bases with the target. Thus, this remodeling might be most effective for unnatural-base aptamers, but could also improve the stability and affinity of some conventional DNA and RNA aptamers.

## Methods

### Oligonucleotides

DNA fragments were purchased from Gene Design or chemically synthesized with an Oligonucleotide Synthesizer nS-8 (Gene Design), using phosphoramidite reagents for the natural and Ds bases (Glen Research). The DNA fragments were purified by gel electrophoresis.

### *T*_m_ measurements

UV melting profiles of aptamers were recorded, using a SHIMADZU UV-2450 spectrometer equipped with a temperature controller (TMSPC-8). The absorbance of each sample (2 μM in 1 mM KH_2_PO_4_, 3 mM Na_2_HPO_4_, and 155 mM NaCl, pH 7.4) was monitored at 260 nm from 15 to 95 °C, at a heating rate of 0.5 °C/min. Each melting temperature was calculated by the first derivative of the melting curve, using the IGOR Pro software (WaveMetrics, Inc.).

### Serum stability analyses

Each DNA aptamer (aptamer 49, aptamer 58, aptamer 57 mh, and aptamer 57 mhGC; final concentration: 2 μM, the aptamers were initially dissolved in 1×PBS) was incubated in 96% human serum (Millipore, Lot #NMM1610699) at 37°C. Aliquots (10 μl) were removed at various time points from 0 to 72 hours, and degradation was terminated by immediate mixing with 110 μl of denaturing solution (1× TBE containing 10 M urea). Each sample was fractionated by 15% denaturing polyacrylamide gel electrophoresis. The DNA was stained with SYBR Gold, detected with a bio-imaging analyzer (Fuji Film LAS-4000), and quantified using the Multi Gauge software to determine the intact fraction. The bands at the top (well positions) on the gel result from the serum, as shown in the lanes without DNA (for only human serum) in Supplementary Fig. 1.

### Binding analyses

Binding affinities of DNA aptamers were examined by SPR measurements, using a BIAcore T200 (GE Healthcare) at 25 °C. Each biotinylated DNA aptamer was diluted to 25 nM in phosphate buffer (1× PBS; 1 mM KH_2_PO_4_, 3 mM Na_2_HPO_4_ and 155 mM NaCl, pH 7.4, Gibco), denatured at 95 °C, cooled slowly to room temperature, and then diluted to 0.5 nM in running buffer (1 × PBS supplemented with 50 mM NaCl (final NaCl concentration: 205 mM), with 0.05% (vol/vol) Nonidet P-40). To reduce the non-specific binding of the protein, such as to the solid-phase support for the measurements, we measured the *K*_D_ values with a relatively high NaCl concentration (Supplementary Fig. 4). Immobilization on a Sensor chip SA (GE Healthcare) was performed by injecting the DNA solution for 8 min, at a flow rate of 5 μl min^−1^ in running buffer. The interaction between the immobilized DNA aptamer and recombinant human IFNγ (Peprotech) was detected by monitoring injections of 1.25 nM, 2.5 nM, 5 nM, 10 nM, 20 nM, 30 nM, and 50 nM IFNγ solutions (diluted with running buffer) in the Kinetic Injection mode. Measurement conditions: flow rate 100 μl min^−1^, protein injection time 150 sec, and dissociation time 450 sec. After each injection, the sensor surface was regenerated with a 5-μl injection of 50 mM NaOH, and the subsequent refolding of the DNA aptamer was accomplished by equilibration with running buffer for 10 min. To determine the *K*_D_ values, control sensorgrams of both a reference cell lacking immobilized DNA fragments on the sensor surface and a measurement with buffer injection were subtracted from each sensorgram of the aptamers, to cancel bulk effects on the sensor chip and response values attributed to nonspecific adsorption, and the data were fitted with a 1 : 1 binding model and a heterogeneous ligand model, using the BIAevaluation T200 software, version 1.0 (GE Healthcare).

### Cell lines and IFNγ stimulation

The human breast cancer cell line, MDA-MB-231, was maintained in Dulbecco’s minimal essential medium (DMEM) (Corning cellgro), supplemented with 10% fetal calf serum (FCS, Atlanta Biologicals Premium Select Heat inactivated at 56°C, Cat#S11550H Lot # H12123) and 1 × L-glutamine, penicillin, and streptomycin (Gibco). To prepare MDA-MB-231 cells for stimulation, the cells (1 × 10^6^ cells/ml in DMEM/10% FCS) were incubated in polystyrene round tubes at 37 °C for 15 min. The cells were centrifuged at 1,200 × g for 5 min, and resuspended in DMEM/10% FCS containing 2 ng/ml IFNγ. The cells were stimulated for 10 min at 37 °C, centrifuged, washed once in PBS to stop the reaction, and prepared for FACS analysis.

### IFNγ stimulation in the presence of aptamers

Aptamers were diluted in PBS to a concentration of 10 μg/ml (final volume: 200–300 μl), heated at 70 °C for 3 min, and then cooled on ice for 1 to 2 min. Before IFNγ stimulation, 50–200 ng/ml aptamer (pre-incubated in DMEM/10% FCS at 37 °C overnight or not) and 2 ng/ml IFNγ were incubated together at 37 °C for 10 min in DMEM/10% FCS. This mixture was then added to the MDA-MB-231 cells, as described above.

### Preparation for FACS Analysis

After stimulation and washing, the cells were resuspended in 1 ml of 2% paraformaldehyde (Electron Microscopy Sciences) for 10 min at room temperature, centrifuged, and resuspended in 1 ml of ice-cold 90% methanol. The cells were incubated for 30 to 60 min or up to 72 h (see Supplementary Fig. 4) at 4 °C in the dark. After centrifugation, the cells were washed twice with 500 μl FACS buffer (1 × PBS pH 7.4 with 0.1% sodium azide and 0.1% BSA), incubated in 100 μl of FACS buffer containing 7.5 μl of anti-P-Stat-1 (BD Phosflow PE mouse anti-Stat-1 pY701) for 30 to 60 minutes at 4 °C in the dark, and washed twice with 500 μl of FACS buffer. The cells were resuspended in 500 μl FACS buffer and analyzed by flow cytometry (De Novo software, FCS Express, version 3).

## Additional Information

**How to cite this article**: Matsunaga, K. *et al.* Architecture of high-affinity unnatural-base DNA aptamers toward pharmaceutical applications. *Sci. Rep.* **5**, 18478; doi: 10.1038/srep18478 (2015).

## Supplementary Material

Supplementary Information
