# Supplementary material for: Architecture of high-affinity unnatural-base DNA aptamers toward pharmaceutical applications
Source: Sci Rep. 2015 Dec 22;5:18478. doi: 10.1038/srep18478 (PMC4686876; doi:10.1038/srep18478)
Supplement: Supplementary Information [file srep18478-s1.pdf]

Supplementary Information

Architecture of high-affinity unnatural-base DNA aptamers toward pharmaceutical applications

Ken-ichiro Matsunaga[1,2,3], Michiko Kimoto[1,2,3,4], Charlotte Hanson[5], Michael Sanford[5], Howard A. Young[5,*], and Ichiro Hirao[1,2,3,*]

[1]*Institute of Bioengineering and Nanotechnology, 31 Biopolis Way, The Nanos, #04-01, Singapore 138669,* [2]*TagCyx Biotechnologies, 1-6-126 Suehiro-cho, Tsurumi-ku, Yokohama, Kanagawa 230-0045, Japan,* [3]*RIKEN Center for Life Science Technologies, 1-7-22 Suehiro-cho, Tsurumi-ku, Yokohama, Kanagawa 230-0045, Japan,* [4]*PRESTO, JST, Honcho, Kawaguchi-shi, Saitama 332-0012, Japan,* [5]*Laboratory of Experimental Immunology, Cancer and Inflammation Program, National Cancer Institute at Frederick, Frederick, MD, 21702 USA*

**Correspondence should be addressed to I. H. (ichiro@ibn.a-star.edu.sg) or H. A. Y. (YoungHow@mail.nih.gov)*

Supplementary Table 1. Oligonucleotide sequences used in this study. The mini-hairpin DNA sequence is red and underlined. The Ds positions and the substitutions from Ds to A are shown in red. The G–C pairs in aptamer 57mhGC that replaced the A–T pairs in aptamer 57mh are shown in blue.

Name	Sequence
Aptamer 49	5′-TCCACTCGGGTCATTTA**Ds**TAATGTAGGT**Ds**TGGGCTAGGC**Ds**GCTAGTGGA-3′
Aptamer 49A	5′-TCCACTCGGGTCATTTA**A**TAATGTAGGT**A**TGGGCTAGGC**A**GCTAGTGGA-3′
Aptamer 26	5′-GGGGTTGGTTGTGTTGGGTGTTGTGT-3′
Aptamer 58	5′-TCCACTCGGGTCATTTA**Ds**TAATGTAGGT**Ds**TGGGCTAGGC**Ds**GCTAGTGGACGCGAAGCG-3′
Aptamer 57mh	5′-TCCACTCGGGTCCGCGAAGCGGTAGGT**Ds**TGGGCTAGGC**Ds**GCTAGTGGACGCGAAGCG-3′
Aptamer 57mhGC	5′-**C**CC**G**C**C**CGGGTCCGCGAAGCGGTAGGT**Ds**TGGGCTAGGC**Ds**GCT**G**G**C**GG**G**CGCGAAGCG-3′

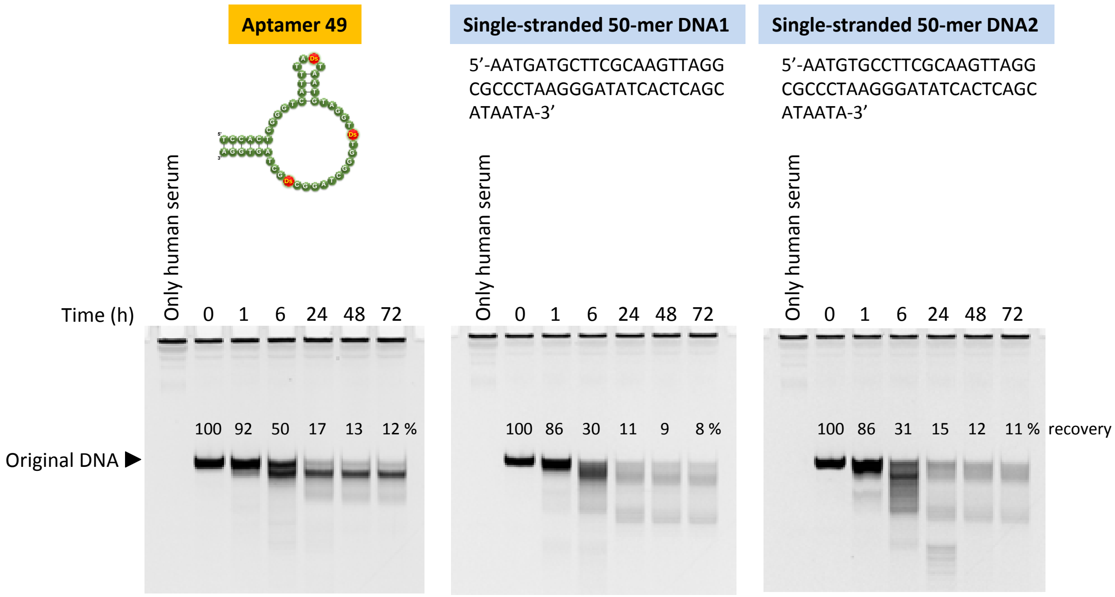

Supplementary Fig. 1. Stability analysis of aptamer 49 and control DNAs in human serum.

Each DNA aptamer (5 μl, aptamer 49, aptamer 58, aptamer 57mh, and aptamer 57mhGC; 50 μM) or Control DNA (5 μl, ssDNA1 and ssDNA2: 50 μM) was mixed with human serum (120 μl), and the mixture (2 μM DNA in 96% serum) was incubated at 37°C. Aliquots (10 μl) were removed at various time points from 0 to 72 hours, and mixed with 110 μl of denaturing solution (1× TBE containing 10 M urea) immediately. Each sample was analyzed by 15% denaturing polyacrylamide gel electrophoresis. DNA stained with SYBR Gold was detected with a bio-imaging analyzer (Fuji Film LAS-4000) and quantified using the Multi Gauge software to determine the intact fraction. The analysis of each sample was repeated twice, and one of them is displayed in this figure.

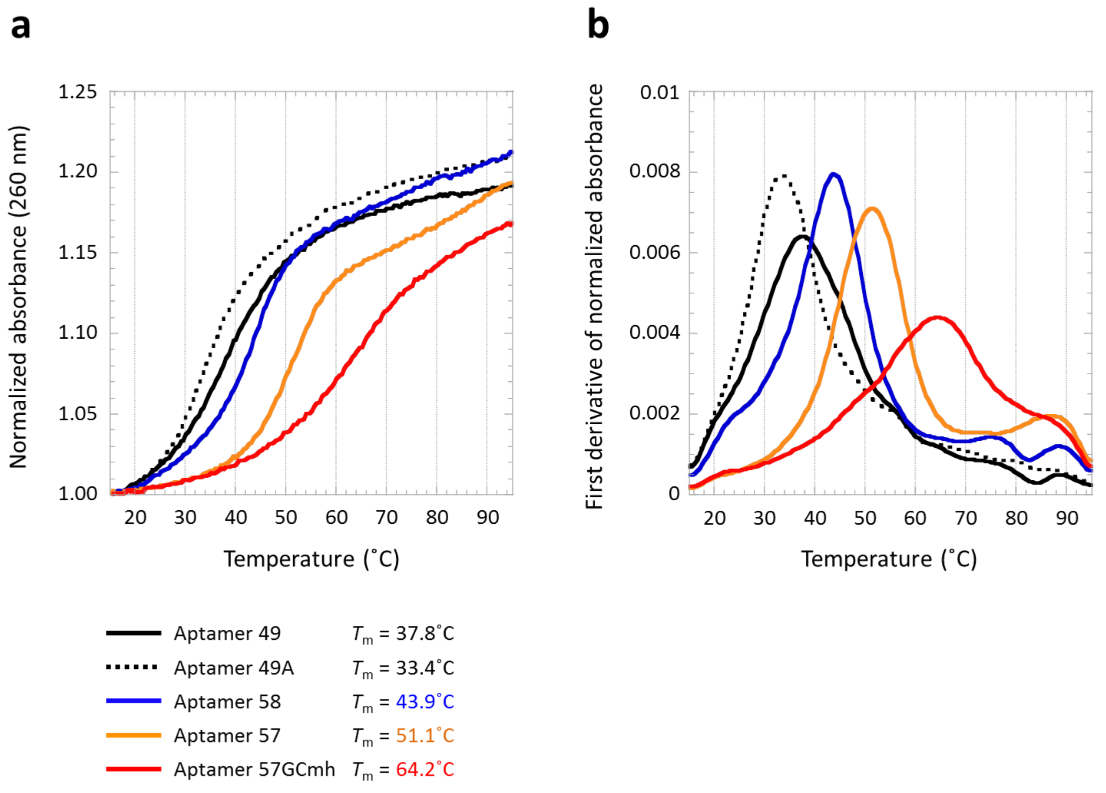

Supplementary Fig. 2. Thermal stabilities of DNA aptamers and their derivatives. UV melting profiles of aptamer 49 (black, solid line), aptamer 49A (black, dotted line), aptamer 58 (blue, solid line), aptamer 57 (orange, solid line), and aptamer 57GCmh (red, solid line) were recorded, using a SHIMADZU UV-2450 spectrometer equipped with a temperature controller (TMSPC-8). The absorbance of each sample (2 μM in 1 mM KH_2PO_4, 3 mM Na_2HPO_4, and 155 mM NaCl, pH 7.4) was monitored at 260 nm from 15 to 95°C, at a heating rate of 0.5°C/min. Each melting temperature was calculated by the first derivative of the melting curve, using the IGOR Pro software (WaveMetrics, Inc.). The melting profiles, normalized by the absorbance at 15°C (left panel), and the first derivatives of the absorbance (right panel) are shown.

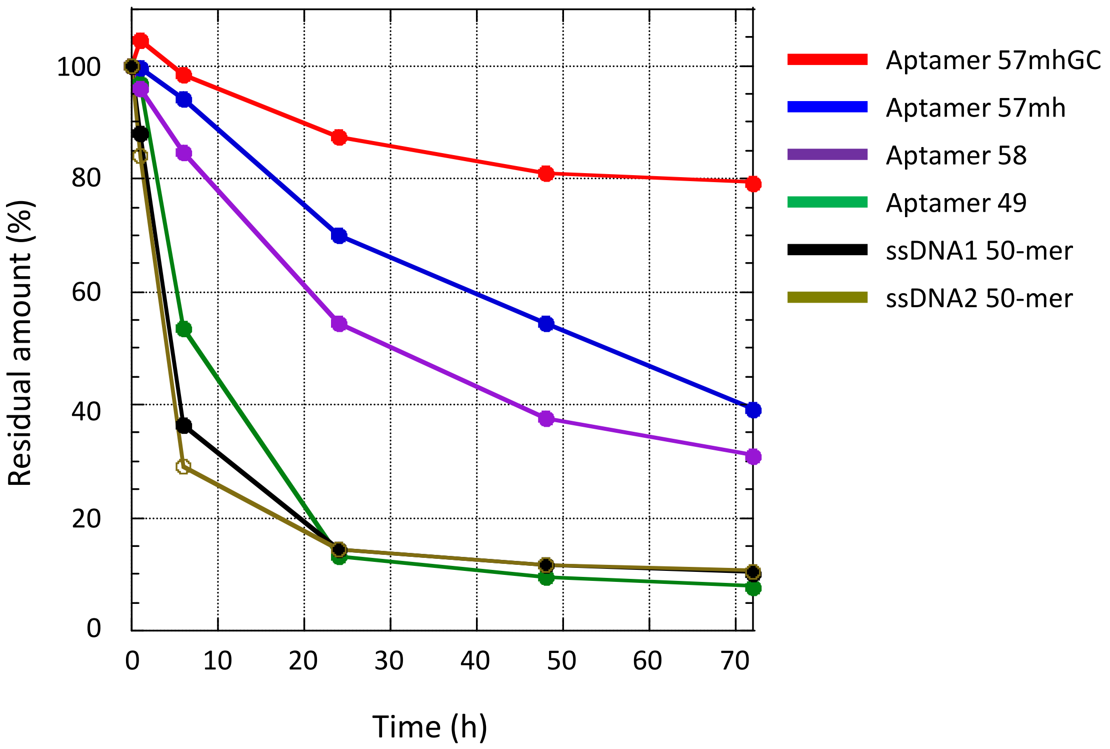

Supplementary Fig. 3. Stability analysis of DNA aptamer and control DNA in human serum.

The graph showed residual amount (%), aptamer 49 (N=4), aptamer 58 (N=2), aptamer 57mh (N=2), aptamer 57mhGC (N=2), ssDNA1 50-mer and ssNDA2 50-mer (N=2). Each DNA aptamer (5 μl, aptamer 49, aptamer 58, aptamer 57mh, and aptamer 57mhGC; 50 μM) or Control DNA (5 μl, ssDNA1 and ssDNA2: 50 μM) was mixed with human serum (120 μl), and the mixture (2 μM DNA in 96% serum) was incubated at 37°C. Aliquots (10 μl) were removed at various time points from 0 to 72 hours, and mixed with 110 μl of denaturing solution (1× TBE containing 10 M urea) immediately. Each sample was analysed by 15% denaturing polyacrylamide gel electrophoresis. DNA stained with SYBR Gold was detected with a bio-imaging analyzer (Fuji Film LAS-4000) and quantified using the Multi Gauge software to determine the intact fraction.

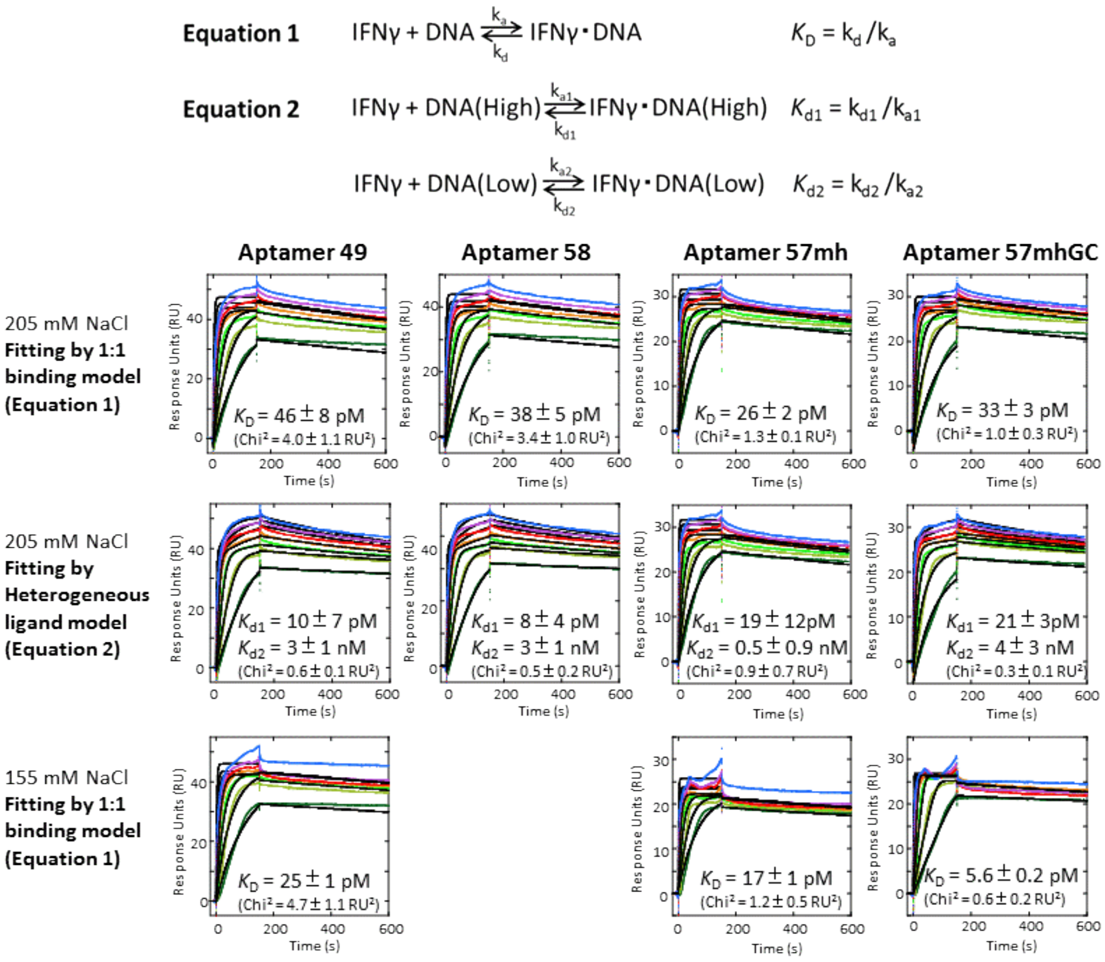

Supplementary Fig. 4. SPR analysis of each DNA aptamer variant against IFNγ in different running buffer and curve fitting with two types of binding model.

The DNA aptamer immobilization and monitoring of IFN-γ binding to DNA aptamers were performed as described in the methods. Running buffer is 1× PBS (1 mM KH_2PO_4, 3 mM Na_2HPO_4 and 155 mM NaCl, pH 7.4, Gibco) supplemented with 0.05% (vol/vol) Nonidet P-40, in the presence or absence of the additional 50 mM NaCl (205 mM or 155 mM NaCl at the final concentration). The data were fitted with a 1:1 binding model (Equation 1) or heterogeneous ligand model (Equation 2) as indicated left on each sensorgram column, by using the BIAevaluation T200 software, version 1.0 (GE Healthcare). In heterogenous ligand model, the K_D values of predominant tight binding were indicated as K_{D1}, and minor binding (weak binding) as K_{D2}. Fitting curves are plotted in black lines. The dissociation constants and chi square values, obtained from each curve fitting, were shown as the average with standard deviations with three data sets.

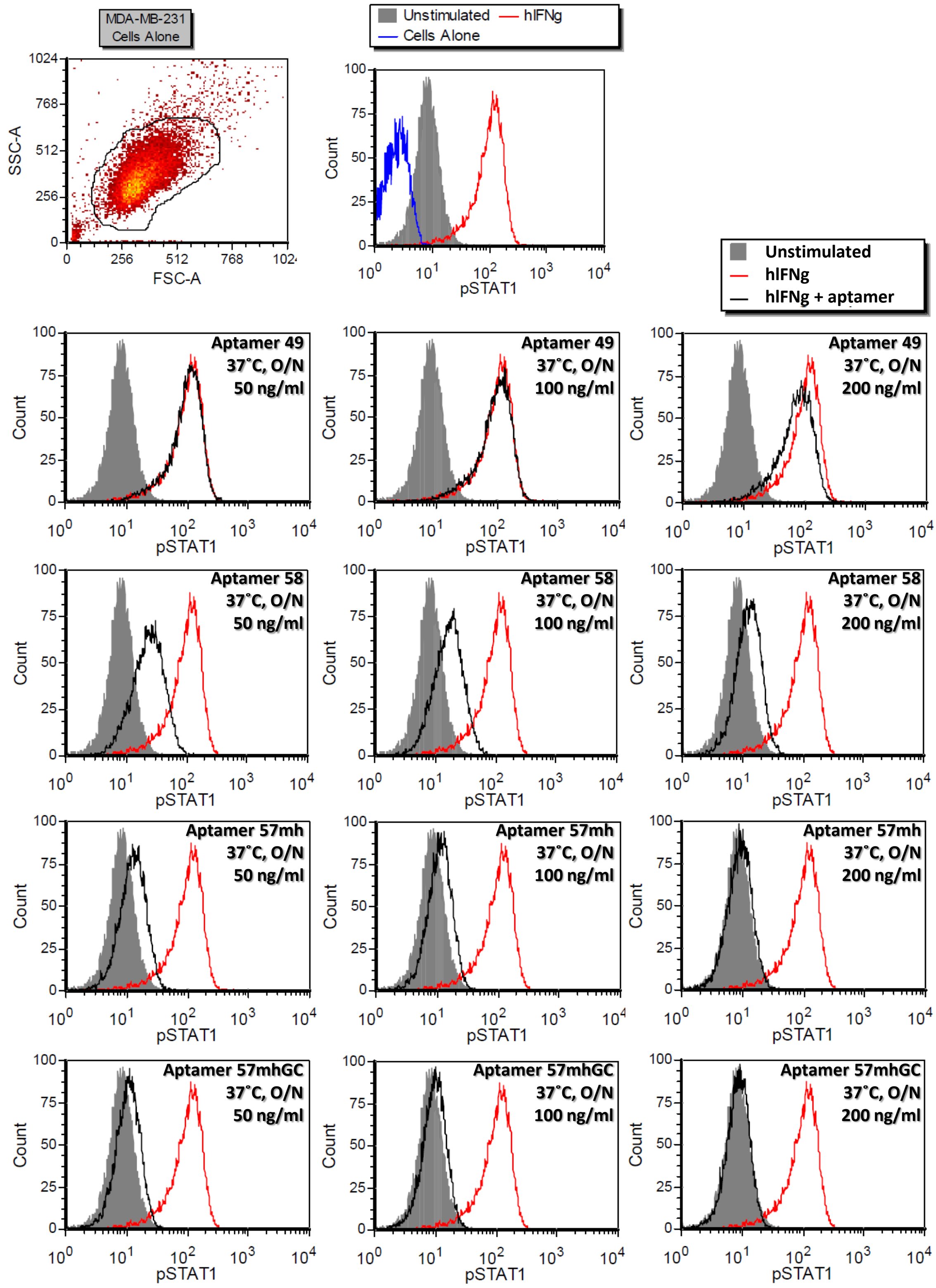

Supplementary Fig. 5. Ability of unnatural-base DNA aptamers (50, 100, and 200 ng/ml) to neutralize human IFNγ (2 ng/ml) induction for STAT1 phosphorylation at 37°C, overnight.

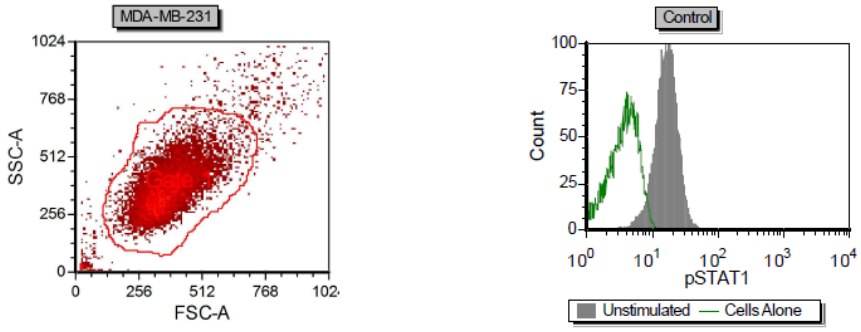

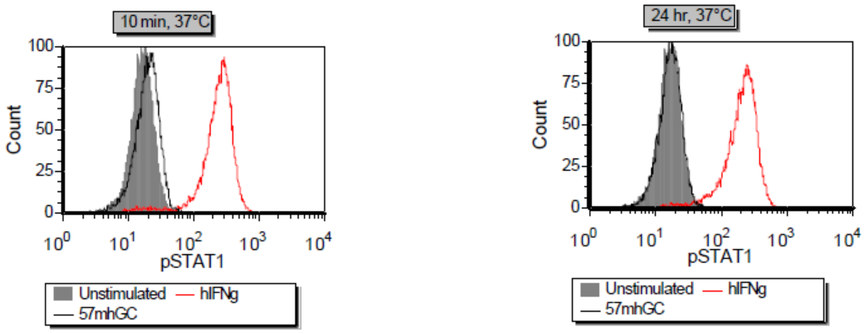

Histogram #	# of Events	Geometric Mean	% Positive	Standard Deviation
1	10041	16.1	0.0	7.3
2	10058	214.8	94.9	119.7
3	10074	18.9	20.1	8.6

Histogram #	# of Events	Geometric Mean	% Positive	Standard Deviation
1	10041	16.1	0.0	7.3
2	10042	188.5	95.7	108.1
3	10059	16.5	3.7	12.7

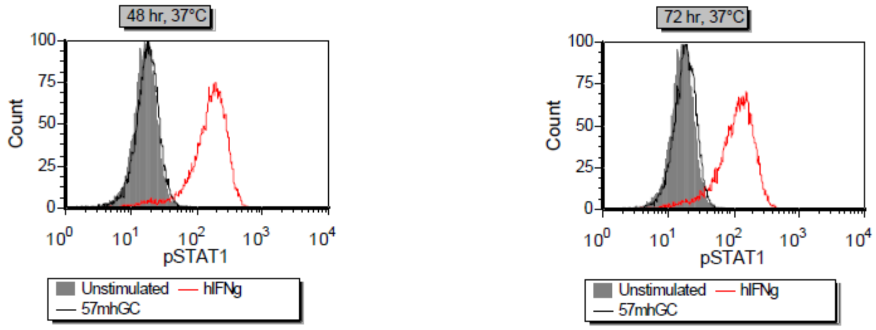

Histogram #	# of Events	Geometric Mean	% Positive	Standard Deviation
1	10041	16.1	0.0	7.3
2	10033	146.4	93.4	93.1
3	10052	17.3	8.6	8.2

Histogram #	# of Events	Geometric Mean	% Positive	Standard Deviation
1	10041	16.1	0.0	7.3
2	10032	103.7	90.7	70.9
3	10040	17.8	11.0	8.7

Supplementary Fig. 6. Ability of aptamer 57GCmh (200 ng/ml) to neutralize human IFNγ (2 ng/ml) induction for STAT1 phosphorylation over a 72-hour time course (10 min, 24 h, 48 h, and 72 h).
